# Application of an Amyloid Beta Oligomer Standard in the sFIDA Assay

**DOI:** 10.3389/fnins.2016.00008

**Published:** 2016-01-29

**Authors:** Katja Kühbach, Maren Hülsemann, Yvonne Herrmann, Kateryna Kravchenko, Andreas Kulawik, Christina Linnartz, Luriano Peters, Kun Wang, Johannes Willbold, Dieter Willbold, Oliver Bannach

**Affiliations:** ^1^ICS-6 Structural Biochemistry, Forschungszentrum Jülich GmbHJülich, Germany; ^2^Institut für Physikalische Biologie, Heinrich-Heine-Universität DüsseldorfDüsseldorf, Germany

**Keywords:** Alzheimer's disease, amyloid-β peptide, diagnostic biomarker, early diagnosis, sFIDA, surface-based fluorescence intensity distribution analysis, stabilized oligomers, standard molecule

## Abstract

Still, there is need for significant improvements in reliable and accurate diagnosis for Alzheimer's disease (AD) at early stages. It is widely accepted that changes in the concentration and conformation of amyloid-β (Aβ) appear several years before the onset of first symptoms of cognitive impairment in AD patients. Because Aβ oligomers are possibly the major toxic species in AD, they are a promising biomarker candidate for the early diagnosis of the disease. To date, a variety of oligomer-specific assays have been developed, many of them ELISAs. Here, we demonstrate the sFIDA assay, a technology highly specific for Aβ oligomers developed toward single particle sensitivity. By spiking stabilized Aβ oligomers to buffer and to body fluids from control donors, we show that the sFIDA readout correlates with the applied concentration of stabilized oligomers diluted in buffer, cerebrospinal fluid (CSF), and blood plasma over several orders of magnitude. The lower limit of detection was calculated to be 22 fM of stabilized oligomers diluted in PBS, 18 fM in CSF, and 14 fM in blood plasma.

## Introduction

Worldwide 5–7% of people older than 60 years are affected by dementia, with Alzheimer's disease (AD) being the most common type. Due to the aging population, the total number of demented people is predicted to increase even further (Prince et al., 2013). There is neither a cure nor a sufficiently reliable laboratory diagnostic test available for this fatal neurodegenerative disease (Lansdall, 2014). Early diagnosis of AD, however, is of great importance for the development of therapeutics and their future application at an early stage of the disease. It is believed that AD can be treated most effectively in preclinical stages, before cognitive functions become impaired and neurons and synapses are damaged irreversibly (Golde et al., 2011). Hitherto, the definitive diagnosis can only be made after the patients' death based on neuropathological hallmarks, like amyloid plaques, neurodegeneration and neurofibrillary tangles (Ballard et al., 2011).

The main component of amyloid plaques is amyloid β peptide (Aβ), which is formed from the amyloid precursor protein (APP) by β- and γ-secretases (Haass et al., 2012). Once released from the precursor, the Aβ peptide is prone to aggregation and can assemble into oligomeric structures and amyloid fibrils. It is widely accepted that soluble Aβ oligomers but not monomers are highly neurotoxic and that the pathological process in AD starts already years before the onset of clinical manifestation (Braak and Braak, 1991; McLean et al., 1999; Cleary et al., 2004; Lesné et al., 2006). Currently, the total concentration of Aβ_42_ in cerebrospinal fluid (CSF), which is lower in AD patients compared to healthy persons (Sunderland et al., 2003; Shaw et al., 2009), is used as a biomarker in clinical trials or academic settings to increase the accuracy of AD diagnosis. At the current stage of biomarker development, however, the total concentration of Aβ_42_ in CSF, even in combination with other biomarkers such as tau protein, does not allow a clear distinction of AD patients from healthy controls or patients with other dementias (Humpel, 2011). Therefore, the development of more accurate biomarkers is of utmost importance.

**Figure 1:**
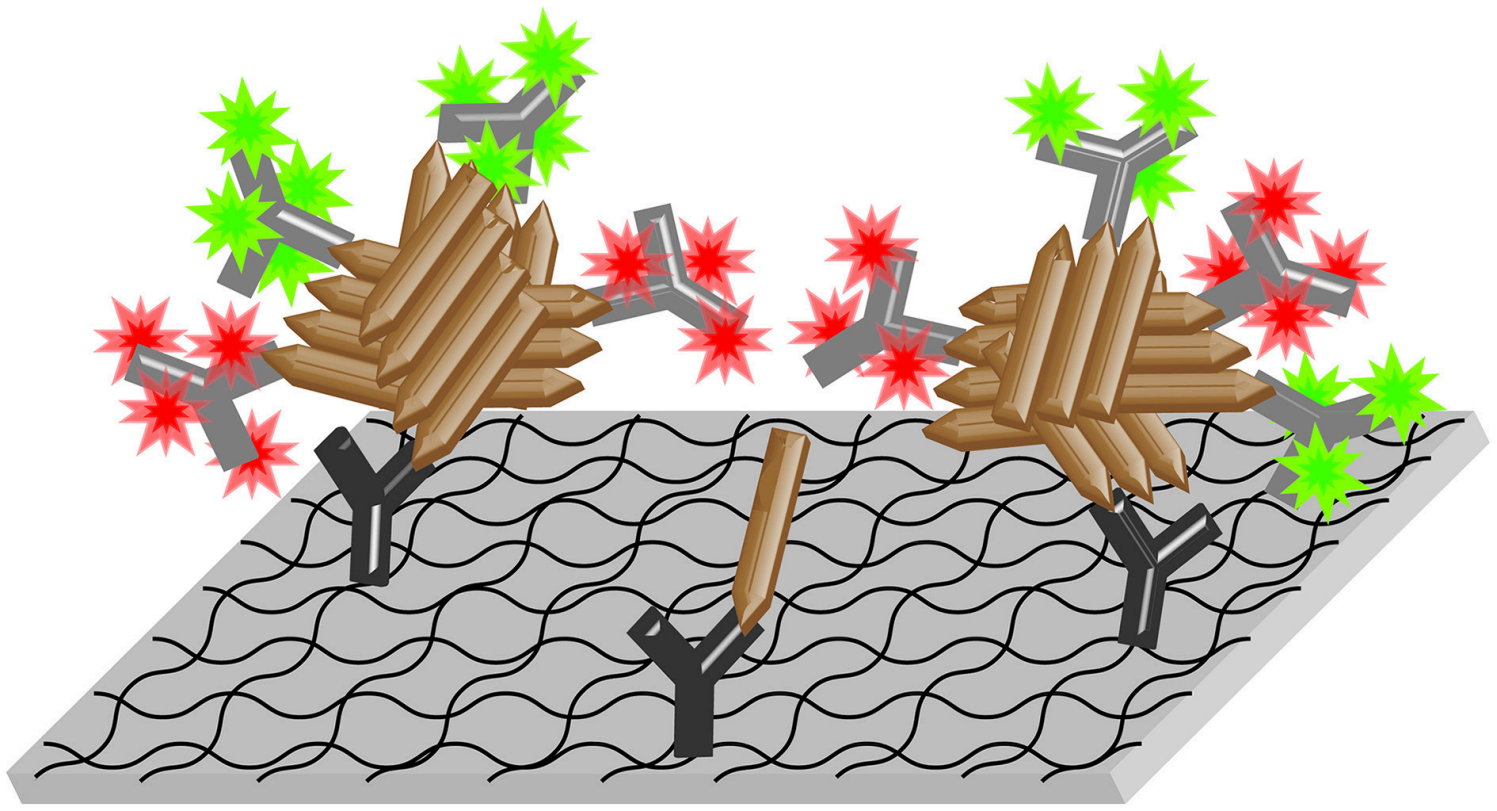
**Scheme of the sFIDA assay**. Aβ-specific capture antibodies (dark gray Y symbols) are immobilized on a functionalized glass surface. Aβ oligomers (brown rods) present in the sample bind to the capture antibodies and are detected by fluorescence labeled (colored stars) anti-Aβ-antibodies (light gray Y symbols). The surface is then imaged by dual-color microscopy. In this version of the assay, all three of the applied antibodies (one capture and two different detection antibodies labeled with two different fluorochromes) bind to overlapping epitopes at the N-terminus of Aβ, which corresponds to the spiky ends of the brown rods in the scheme above. Thereby, only oligomers with multiple epitopes, but not monomers, are able to bind detection antibodies while bound to the capture and thus yield detectable signals.

Since Aβ oligomeric species are known to be directly involved in AD pathology or even to trigger the disease (Haass and Selkoe, 2007), Aβ oligomers are considered as promising biomarker for AD (Blennow et al., 2010). The main challenges for Aβ oligomer-based diagnostics in body fluids are the presumably very low concentrations of Aβ oligomers and the high background of monomeric Aβ (Rosén et al., 2013). To meet those requirements, we have previously developed an assay called sFIDA (surface-based fluorescence intensity distribution analysis; Birkmann et al., 2007; Funke et al., 2007, 2010; Bannach et al., 2012). The principle of sFIDA is illustrated in Figure 1. The biochemical setup of sFIDA resembles a conventional sandwich ELISA. All Aβ species are immobilized on a functionalized glass surface via Aβ-specific capture antibodies. After immobilization, Aβ aggregates are multiply loaded by at least two detection antibodies, each of them labeled with a different fluorochrome. Because capture and detection antibodies recognize the same or an overlapping epitope on Aβ, Aβ monomers cannot bind any detection antibodies while bound to the capture antibody. In contrast to a classical ELISA, the result of the measurement is not a single readout for the whole sample. Instead, the surface is imaged by high-resolution fluorescence microscopy, such as dual-color total internal reflection fluorescence microscopy (TIRFM). Only those pixels that show signal intensities above the background noise in both channels are counted. Thus, the number of colocalized pixels above the background noise is expected to correlate with the concentration of Aβ oligomers in the sample.

Results showing increased sFIDA readouts for AD patients compared to non-demented controls have been reported previously (Wang-Dietrich et al., 2013). However, in this study no reliable Aβ oligomer standard was available to determine absolute concentrations from the assay readout. Due to both the dynamic aggregation and dissociation of Aβ, non-stabilized oligomers are not suited as standard in oligomer-based diagnostic assays.

Here, we demonstrate application of stabilized Aβ oligomers as standard molecules in the sFIDA assay. The sFIDA readout correlates with the applied oligomer concentration over five magnitudes down to a femtomolar range, which will allow the quantification of natural Aβ oligomer concentrations in body fluids.

## Materials and methods

### Biological samples and PBS spiked with stabilized oligomers

Four individual human EDTA-anticoagulated plasma samples (Zen-Bio, Research Triangle Park, USA) and one pooled human EDTA-anticoagulated plasma sample from three healthy donors were centrifuged for 15 min at 15,000 × *g*. The supernatant was collected and equal volumes from each sample were combined to obtain one large pool from several donors (from here on referred to as “plasma fraction”). Human cerebrospinal fluid sample (CSF; pooled from healthy donors/mixed gender) was purchased from Biochemed (Winchester, USA). Stabilized Aβ oligomers (Crossbeta Biosciences B.V., Utrecht, the Netherlands), from here on called “oligomers”, were serially diluted from the stock solution (10 nM) to concentrations of 1 nM, 100 pM, 10 pM, 1 pM, 100 fM, 10 fM, and 1 fM in PBS (GE Healthcare, Chalfont St. Giles, UK), CSF or the plasma fraction as described above. All concentrations of Aβ oligomers in this publication refer to oligomer particle concentrations, if not stated otherwise. The oligomers consist of approximately 220 Aβ_1–42_ monomers (manufacturer's data); further characterization of the stabilized oligomers, including data on the size homogeneity and stability, are available on the manufacturer's homepage (Crossbeta Biosciences, 2015).

### sFIDA

#### Plate preparation

384-well plates (SensoPlate Plus with 175 μm glass bottom; Greiner Bio-One, Kremsmünster, Austria) were used for sFIDA. Functionalization of the glass surface was performed as previously described in Janissen et al. (2009). The surface was treated with 5 M NaOH (AppliChem, Darmstadt, Germany) for 15 min, washed three times with water, neutralized with 1 M HCl (AppliChem, Darmstadt, Germany; 15 min), washed again three times with water and then twice with 70% ethanol (VWR International, Langenfeld, Germany). After drying the plate at room temperature, the wells were incubated in 10 M ethanolamine in DMSO (Sigma-Aldrich, St. Louis, USA) overnight. Afterwards, the wells were washed three times with DMSO, twice with 70% ethanol and the plate was dried again at room temperature. A solution of 50 mM SC-PEG-CM (MW 5000 Da, Laysan Bio, Arab, USA) in DMSO was heated shortly to 70°C until the PEG dissolved. After the solution cooled down, 2% (v/v) triethylamin (Fluka, Buchs, Switzerland) were added, the solution was quickly vortexed and 15 μl were applied per well. After an incubation time of 1 h the wells were washed five times with water.

The carboxymethyl groups of SC-PEG-CM on the glass surface were then activated by addition of 30 μl of 100 mM EDC (Fluka, Buchs, Switzerland)/100 mM NHS (Aldrich, Milwaukee, USA) in 0.1 M MES buffer, pH 3.5 (AppliChem) per well for 30 min. After flushing the wells three times quickly with MES buffer, 15 μl of 10 ng/μl capture antibody Nab228 in PBS (the supernatant after centrifuging 10 min at 18,000 g) was added to the surface. After incubating for 90 min, unbound antibody was removed and wells were washed three times with PBST (PBS + 0.05% Tween20, AppliChem Panreac, Darmstadt, Germany) and three times with PBS. Then 50 μl of blocking solution (SmartBlock, CANDOR Biosciences, Wangen, Germany) per well were incubated for 1 h. After washing the wells three times with PBST and three times with PBS, 15 μl sample was applied to each well and incubated overnight. The wells were washed once with PBST and twice with PBS. The detection antibodies 6E10 labeled with Alexa Fluor 488 (Covance, Princeton, USA) and Nab228 labeled with Alexa Fluor 647 (Santa Cruz, Dallas, USA) were combined to each 1.25 μg/ml in PBS and centrifuged for 1 h (100,000 g, 4°C). The supernatant was mixed and added to the wells (15 μl/well, 1 h). Finally, the wells were washed once with PBST and twice with PBS. The buffer was removed and 100 μl of water were applied to each well for image acquisition on TIRFM (AM TIRF MC, Leica microsystems, Wetzlar, Germany).

#### Image data acquisition

Using TIRF microscopy, 25 positions per well were imaged in two different channels (14 bit gray scale; channel 0: excitation at 635 nm, emission filter 705/72 nm; channel 1: excitation at 488 nm, emission filter 525/36 nm). Each image contains 1000 × 1000 pixels and represents an area of 116 × 116 μm. In total, 3.15% of the well surface was imaged.

#### Image data analysis

Prior to data analysis, images showing inhomogeneous surfaces, e.g., due to mechanical damage of the surface or impurities, were excluded from the analysis by automated artifact detection, which is briefly described in the following: Each original image was converted to a binary image by replacing all pixels having intensities above or equal the mean pixel intensity of the regarding image plus one standard deviation with the number one, all others with the number zero. In the next step, erosion was applied to these binary images by using a rectangular structuring element with a size of 31 × 31 pixels. After erosion, the binary image was dilated using the same structuring element as for erosion. Each cluster that consisted of connected pixels with the intensity one in the binary image after dilatation was then analyzed in the original image. Clusters showing either a mean pixel intensity of above 4000, a standard deviation of pixel intensities above 2800, or a skewness of <0 in the original images were defined as artificial and the whole image was excluded from the analysis. Images that had a mean pixel intensity of 16,383 over the whole image in at least one channel were included for image analysis although they were excluded by the artifact detection, because those images are estimated as being saturated, but not artificial.

To account for inhomogeneous illumination, only the central “region of interest” containing 500 × 500 pixels of each image were used for further analysis.

The remaining images were analyzed for colocalization: For both channels, intensity cutoffs for exclusion of background signal were determined. As the background signal might differ from one matrix (i.e., PBS, CSF, and plasma fraction) to another, the cutoff values were determined for each matrix individually, but—in order to compare sFIDA readouts achieved by diluting oligomers in the different matrices—in a reliable and unbiased way. The cutoff for each channel and each matrix was determined from the unspiked control sample to be the value, which is exceeded by only 0.01% of total image pixels. This value represents a reasonable compromise between efficient background removal and retention of assay sensitivity. For cutoffs used in this study, see Table 1.

**Table 1:** **Cutoffs for the different body fluids**.

**Matrix**	**Cutoff channel 635 nm**	**Cutoff channel 488 nm**
CSF	3268	2339
PBS	4082	2773
Plasma fraction	4259	2028

Cutoffs were obtained for each channel and matrix by allowing only 0.01% of all pixels to be above background signal for negative controls.

Colocalized pixels with intensity values above the cutoffs in both channels were counted for each image. The number of colocalized pixels was determined for each picture and the average pixel count from all pictures from the same sample was referred to as “sFIDA readout”. Please note that the sFIDA readout cannot exceed 250,000, which corresponds to the total number of pixels per analyzed image section.

### Calculation of calibration curves

For the calibration of assay readout (number of colocalized pixels) to molecule concentration a weighted linear regression analysis was performed with Matlab (The MathWorks, Natick, USA) from experimental data points within the linear detection range (CSF: 100 pM to 10 fM; PBS: 10 pM to 10 fM; plasma fraction: 10 pM to 10 fM) with respective weights calculated as 1/readout. In cases of readout = 0 the weight was determined as 1.

### Statistics

In order to statistically assess differences between sFIDA readouts of different concentrations of oligomers diluted in the same matrix, two-way omnibus Kruskal-Wallis test was used for comparison of more than two groups. *Post-hoc* analysis was performed by using two-tailed Mann-Whitney-U test and *p*-value adjustment according to Benjamini and Hochberg (1995) in order to account for multiple testing. By Mann-Whitney-U test, sFIDA readouts from each concentration were compared to the next lower one. Additionally, sFIDA readouts from blank samples were compared to readouts from 10 to 100 fM. The false discovery rate controlling procedure after Benjamini and Hochberg was calculated for 0.05 (for significant results, indicated with ^*^) and 0.01 (for very significant results, indicated with ^**^). Kruskal-Wallis and Mann-Whitney-U test were calculated using the statistical software Origin (OriginLab Corporation, Northampton, USA), false discovery rate controlling procedure after Benjamini and Hochberg was calculated in Microsoft Excel (Microsoft Corporation, Redmond, USA).

## Results

### Detection of stabilized oligomers by sFIDA

**Figure 2:**
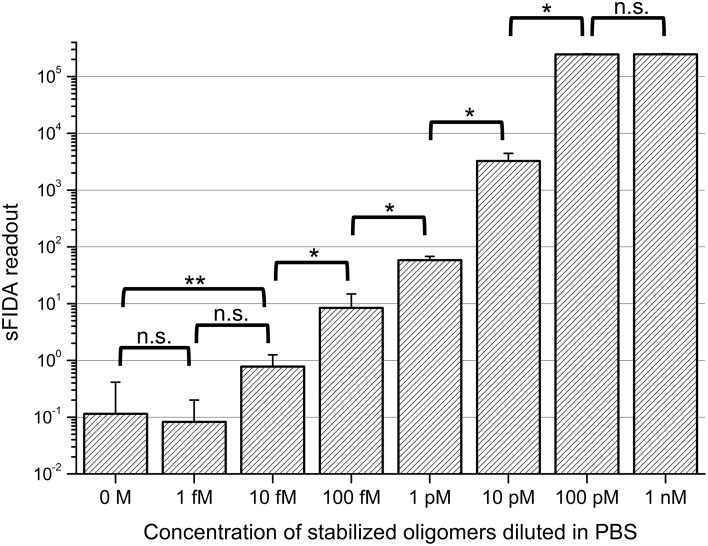
**sFIDA readout of stabilized oligomers diluted in PBS**. Columns and error bars represent the mean values and standard deviations calculated from a fourfold determination of samples containing oligomers. The blank was determined 21-fold. Cutoffs for each channel were set to discard virtually all background from control samples except for 25 pixels, which are 0.01% of all pixels. This led to the following cutoff values (channel 635 nm/channel 488 nm): 4082/2773. Please note that the number of colocalized pixels (sFIDA readout) is lower than the number of pixels above background in the single channels. n.s., not significant; ^*^p ≤ 0.05; ^**^p ≤ 0.01.

In a first set of experiments we sought to find out if the stabilized oligomers can be sensitively detected by the sFIDA assay. Therefore, a log10 dilution series of oligomers in PBS with concentrations ranging from 1 nM to 1 fM was subjected to sFIDA analysis in quadruplicate determination. As can be seen in Figure 2, the sFIDA readout correlated well with the applied concentration of stabilized oligomers in the range of 100 pM down to 1 fM. The readouts from 1 nM to 100 pM oligomers in PBS reached saturation, which means that all pixels were above cutoff in both channels. At the lower end of the dilution series, the sFIDA readout of the lowest oligomer concentration (1 fM) did not differ significantly from the readouts from 10 fM oligomers and the blank control. However, there was a significant difference in the sFIDA readouts from 10 fM oligomers and the blank control.

### Spiking of CSF, PBS, and EDTA plasma fraction with stabilized oligomers

**Figure 3:**
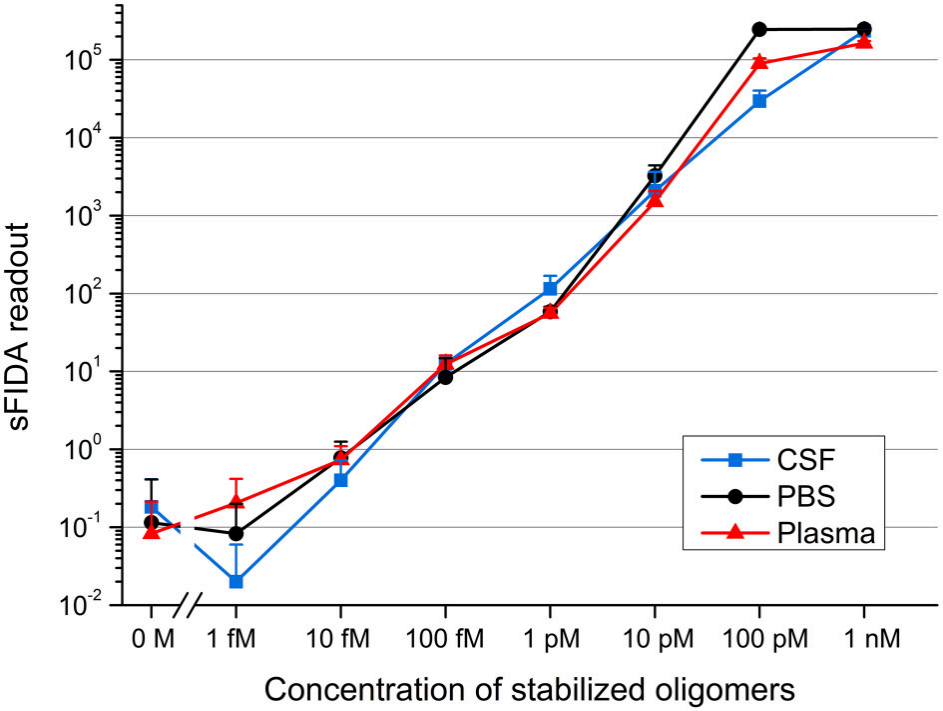
**sFIDA readout of stabilized oligomers diluted in CSF, PBS, and a plasma fraction**. Shown are mean values and standard deviations from fourfold (samples containing stabilized oligomers) or 21-fold (all blanks) determinations. Cutoffs for channel 635 nm/channel 488 nm: CSF, 3268/2339; PBS, 4082/2773; plasma fraction, 4259/2028.

After demonstrating the ability to detect even femtomolar concentrations of stabilized oligomers diluted in buffer, we investigated if different body fluid environments affect the sensitivity of oligomer detection by sFIDA. To check for matrix effects that possibly attenuate the specific signal of Aβ oligomers, the oligomers were spiked into CSF and blood plasma from healthy, non-demented control subjects. All samples containing oligomers were determined fourfold by sFIDA analysis, while each blank sample was measured 21-fold. Figure 3 shows the mean sFIDA readouts for all samples.

The sFIDA readout correlated well with the oligomer concentration down to 1 fM. However, there was no significant difference in the readouts of 10 fM as compared to 1 fM, as well as in the readouts from the blank sample compared to 1 and 10 fM oligomers spiked into CSF. sFIDA readouts from 100 fM and the blank sample differed significantly.

For plasma samples, there was even a very significant difference between the sFIDA readouts of 10 fM and blank sample.

### Lower limits of detection and lower limits of quantification for stabilized oligomers diluted in PBS, CSF, and the plasma fraction

As the concentration of Aβ oligomers in body fluids like CSF and blood is presumably very low (Bruggink et al., 2013; Hölttä et al., 2013; Savage et al., 2014), the lower limit of detection (LLOD) is an important characteristic of every assay for the determination of Aβ oligomer concentration. To identify the LLOD for each matrix used in this report, each blank sample was determined 21-fold. The LLOD was calculated as the mean sFIDA readout from all blank samples plus three times the standard deviation. By establishing a calibration curve from the dilution series, the Aβ oligomer concentration corresponding to the calculated sFIDA readout was then determined. The resulting LLODs were 22 fM for stabilized oligomers diluted in PBS, 18 fM in CSF, and 14 fM in the plasma fraction.

The lower limits of quantification (LLOQ) were calculated as the mean sFIDA readout from all blank samples plus ten times the standard deviation. The same calibration curves as used for determination of LLOD were applied, leading to the following concentrations: 32 fM for stabilized oligomers diluted in PBS, 24 fM for dilution in CSF, and 22 fM for dilution in the plasma fraction.

## Discussion

In the present work we applied stabilized Aβ oligomers as standard in the sFIDA assay. For dilutions in PBS, CSF from control donors, and blood plasma from control donors, the sFIDA readout correlated with the oligomer concentration over five to six orders of magnitude. Although oligomer concentrations in the upper picomolar range are presumably not physiologically relevant, the observed linearity over several orders of magnitude is useful to check assay functionality and to facilitate assay calibration. The calculated LLODs for oligomers diluted in PBS, CSF, and a plasma fraction were in the range of 14–22 fM particle concentration. We can exclude that endogenous Aβ oligomers, which are possibly present also in healthy subjects, contribute significantly to the assay readout, since the intensity cutoff was determined based on the non-spiked control samples.

For the lower concentrations from 1 pM down to 1 fM, a linear relation between the sFIDA readout and concentrations of Aβ oligomers was observed. We expect that to be the relevant concentration range for analysis of biological samples, as published concentrations of oligomers in CSF are in the femtomolar to low picomolar range (stated as monomeric concentrations of Aβ; oligomeric concentrations are even lower; Bruggink et al., 2013; Hölttä et al., 2013; Savage et al., 2014).

LLODs often refer to the concentration or mass of the total applied peptide, although the actual portion of oligomerized Aβ and the size of Aβ oligomers in the preparations is mostly unknown (Santos et al., 2007; Sancesario et al., 2012). The concentration of 14 fM of the stabilized oligomers used in this study corresponds to 3.1 pM (13.9 ng/L) monomeric Aβ_1–42_. The LLOD given in mass per volume is roughly in the same range or above the limits of detection published for some Aβ oligomer specific ELISA (Fukumoto et al., 2010; Bruggink et al., 2013; Hölttä et al., 2013; Savage et al., 2014). In principle, sFIDA allows detection and quantification of single particles of oligomers consisting of approximately 220 Aβ monomers.

Although the stabilized oligomers used in this study might not accurately reflect the properties of native Aβ oligomers in terms of composition, mass, and structural heterogeneity, they are nevertheless a valuable tool for assay development, assay calibration, and determination of inter- and intra-assay variation due to their stability and homogenous size. While heterogeneous Aβ oligomer standards would resemble endogenous conditions more closely, it is hardly possible to reliably produce such standards with minimal batch-to-batch-variations thus limiting their use in assay validation.

The stabilized oligomers are advantageous with regard to long term stability and they can easily be distributed to compare inter-laboratory results. This enables to thoroughly validate and calibrate an assay, which is a very important feature in assay development. However, the applicability of this standard for biological samples will have to be addressed in future studies. Quantification of very small oligomers in body fluids might emphasize the need for even smaller standard oligomers than the ones used in this study.

We have previously shown that monomers of synthetic Aβ give not rise to significant signals in the sFIDA assay by using overlapping epitopes in the capture and detection system (Wang-Dietrich et al., 2013). When analyzing native CSF samples in diagnostic setups, however, experimental conditions (i.e., pH, incubation times, freeze/thaw cycles) have to be carefully adjusted to avoid false-positive signals due to artificial aggregation of endogenous Aβ monomers.

In the present version of the assay, two N-terminal antibodies were used for capturing and detection of Aβ, i.e., Nab228 (epitope Aβ1-11) and 6E10 (epitope Aβ3-8). By using alternative capture and probe antibodies, it is not only possible to detect oligomers composed of different Aβ isoforms, but also to detect hybrid aggregates composed of different peptides or proteins. Therefore, sFIDA assay can in future be applied for scientific purpose in order to investigate the presence and pathological relevance of different oligomeric species in body fluids or brain homogenates of patients with different neurodegenerative diseases, such as AD. Additionally, after thorough investigation and validation of the assay and the measured targets, sFIDA might either give extra information useful for diagnostics or even measure oligomeric biomarkers that allow a reliable diagnosis, and might be useful for disease monitoring in clinical trials during treatment.

## Author contributions

KKU, MH, YH, KKR, AK, CL, LP, KW, JW conducted experiments. KKU, DW, and OB designed experiments and wrote the manuscript.

### Conflict of interest statement

The authors declare that the research was conducted in the absence of any commercial or financial relationships that could be construed as a potential conflict of interest.

## References

[B1] Ballard C. Gauthier S. Corbett A. Brayne C. Aarsland D. Jones E. (2011). Alzheimer's disease. Lancet 377, 1019–1031. 10.1016/S0140-6736(10)61349-9 21371747

[B2] Bannach O. Birkmann E. Reinartz E. Jaeger K. E. Langeveld J. P. Rohwer R. G.. (2012). Detection of prion protein particles in blood plasma of scrapie infected sheep. PLoS ONE 7:e36620. 10.1371/journal.pone.0036620 22567169 PMC3342177

[B3] Benjamini Y. Hochberg Y. (1995). Controlling the false discovery rate: a practical and powerful approach to multiple testing. J. R. Stat. Soc. B 57, 289–300.

[B4] Birkmann E. Henke F. Weinmann N. Dumpitak C. Groschup M. Funke A.. (2007). Counting of single prion particles bound to a capture-antibody surface (surface-FIDA). Vet. Microbiol. 123, 294–304. 10.1016/j.vetmic.2007.04.001 17499942

[B5] Blennow K. Hampel H. Weiner M. Zetterberg H. (2010). Cerebrospinal fluid and plasma biomarkers in Alzheimer disease. Nat. Rev. Neurol. 6, 131–144. 10.1038/nrneurol.2010.4 20157306

[B6] Braak H. Braak E. (1991). Neuropathological stageing of Alzheimer-related changes. Acta Neuropathol. 82, 239–259. 10.1007/BF00308809 1759558

[B7] Bruggink K. A. Jongbloed W. Biemans E. A. L. M. Veerhuis R. Claassen J. A. H. R. Verbeek M. M.. (2013). Amyloid-β oligomer detection by ELISA in cerebrospinal fluid and brain tissue. Anal. Biochem. 433, 112–120. 10.1016/j.ab.2012.09.014 23022042

[B8] Cleary J. P. Walsh D. M. Hofmeister J. J. Shankar G. M. Kuskowski M. A. Selkoe D. J.. (2004). Natural oligomers of the amyloid-β protein specifically disrupt cognitive function. Nat. Neurosci. 8, 79–84. 10.1038/nn1372 15608634

[B9] Crossbeta Biosciences (2015). Crossbeta Oligomer Publications [Online]. Available online at: http://www.crossbeta.com/technology/publications/ (Accessed December 07, 2015).

[B10] Fukumoto H. Tokuda T. Kasai T. Ishigami N. Hidaka H. Kondo M.. (2010). High-molecular-weight β-amyloid oligomers are elevated in cerebrospinal fluid of Alzheimer patients. FASEB J. 24, 2716–2726. 10.1096/fj.09-150359 20339023

[B11] Funke S. A. Birkmann E. Henke F. Görtz P. Lange-Asschenfeldt C. Riesner D.. (2007). Single particle detection of Abeta aggregates associated with Alzheimer's disease. Biochem. Biophys. Res. Commun. 364, 902–907. 10.1016/j.bbrc.2007.10.085 17963690

[B12] Funke S. A. Wang L. Birkmann E. Willbold D. (2010). Single-particle detection system for Aβ aggregates: adaptation of surface-fluorescence intensity distribution analysis to laser scanning microscopy. Rejuvenation Res. 13, 206–209. 10.1089/rej.2009.0925 19961303

[B13] Golde T. E. Schneider L. S. Koo E. H. (2011). Anti-aβ therapeutics in Alzheimer's disease: the need for a paradigm shift. Neuron 69, 203–213. 10.1016/j.neuron.2011.01.002 21262461 PMC3058906

[B14] Haass C. Kaether C. Thinakaran G. Sisodia S. (2012). Trafficking and proteolytic processing of APP. Cold Spring Harb. Perspect. Med. 2:a006270. 10.1101/cshperspect.a006270 22553493 PMC3331683

[B15] Haass C. Selkoe D. J. (2007). Soluble protein oligomers in neurodegeneration: lessons from the Alzheimer's amyloid β-peptide. Nat. Rev. Mol. Cell Biol. 8, 101–112. 10.1038/nrm2101 17245412

[B16] Hölttä M. Hansson O. Andreasson U. Hertze J. Minthon L. Nägga K.. (2013). Evaluating amyloid-β oligomers in cerebrospinal fluid as a biomarker for Alzheimer's disease. PLoS ONE 8:e66381. 10.1371/journal.pone.0066381 23799095 PMC3682966

[B17] Humpel C. (2011). Identifying and validating biomarkers for Alzheimer's disease. Trends Biotechnol. 29, 26–32. 10.1016/j.tibtech.2010.09.007 20971518 PMC3016495

[B18] Janissen R. Oberbarnscheidt L. Oesterhelt F. (2009). Optimized straight forward procedure for covalent surface immobilization of different biomolecules for single molecule applications. Colloids Surf. B Biointerfaces 71, 200–207. 10.1016/j.colsurfb.2009.02.011 19329289

[B19] Lansdall C. J. (2014). An effective treatment for Alzheimer's disease must consider both amyloid and tau. Biosci. Horizons 7:hzu002 10.1093/biohorizons/hzu002

[B20] Lesné S. Koh M. T. Kotilinek L. Kayed R. Glabe C. G. Yang A.. (2006). A specific amyloid-β protein assembly in the brain impairs memory. Nature 440, 352–357. 10.1038/nature04533 16541076

[B21] McLean C. A. Cherny R. A. Fraser F. W. Fuller S. J. Smith M. J. Vbeyreuther K.. (1999). Soluble pool of Aβ amyloid as a determinant of severity of neurodegeneration in Alzheimer's disease. Ann. Neurol. 46, 860–866. 10589538 10.1002/1531-8249(199912)46:6<860::aid-ana8>3.0.co;2-m

[B22] Prince M. Bryce R. Albanese E. Wimo A. Ribeiro W. Ferri C. P. (2013). The global prevalence of dementia: a systematic review and metaanalysis. Alzheimer's Dement. 9, 63–75. 10.1016/j.jalz.2012.11.007 23305823

[B23] Rosén C. Hansson O. Blennow K. Zetterberg H. (2013). Fluid biomarkers in Alzheimer's disease - current concepts. Mol. Neurodegener. 8:20. 10.1186/1750-1326-8-20 23800368 PMC3691925

[B24] Sancesario G. M. Cencioni M. T. Esposito Z. Borsellino G. Nuccetelli M. Martorana A.. (2012). The load of amyloid-β oligomers is decreased in the cerebrospinal fluid of Alzheimer's disease patients. J. Alzheimers Dis. 31, 865–878. 10.3233/JAD-2012-120211 22717612

[B25] Santos A. N. Torkler S. Nowak D. Schlittig C. Goerdes M. Lauber T.. (2007). Detection of amyloid-β oligomers in human cerebrospinal fluid by flow cytometry and fluorescence resonance energy transfer. J. Alzheimers Dis. 11, 117–125. 17361040 10.3233/jad-2007-11114

[B26] Savage M. J. Kalinina J. Wolfe A. Tugusheva K. Korn R. Cash-Mason T.. (2014). A sensitive Aβ oligomer assay discriminates alzheimer's and aged control cerebrospinal fluid. J. Neurosci. 34, 2884–2897. 10.1523/JNEUROSCI.1675-13.2014 24553930 PMC6608513

[B27] Shaw L. M. Vanderstichele H. Knapik-Czajka M. Clark C. M. Aisen P. S. Petersen R. C.. (2009). Cerebrospinal fluid biomarker signature in Alzheimer's disease neuroimaging initiative subjects. Ann. Neurol. 65, 403–413. 10.1002/ana.21610 19296504 PMC2696350

[B28] Sunderland T. Linker G. Mirza N. Putnam K. T. Friedman D. L. Kimmel L. H.. (2003). Decreased β-amyloid1-42 and increased tau levels in cerebrospinal fluid of patients with Alzheimer disease. JAMA 289, 2094–2103. 10.1001/jama.289.16.2094 12709467

[B29] Wang-Dietrich L. Funke S. A. Kühbach K. Wang K. Besmehn A. Willbold S.. (2013). The amyloid-β oligomer count in cerebrospinal fluid is a biomarker for Alzheimer's disease. J. Alzheimers Dis. 34, 985–994. 10.3233/JAD-122047 23313925

